# Supplementary material for: Creation of a shortened version of the Sleep Disorders Questionnaire (SDQ)
Source: PLoS One. 2024 Feb 6;19(2):e0288216. doi: 10.1371/journal.pone.0288216 (PMC10846718; doi:10.1371/journal.pone.0288216)
Supplement: S1 Fig — (DOCX) [file pone.0288216.s001.docx]

**S1 Fig. Scree plots of the subfactors of all 4 major factors**

**S1 Fig., Part A. Scree plot for sub-factors of Main Factor 1, "Insomnia"**

**S1 Fig., Part B. Scree plot for sub-factors of Main Factor 2, "Narcolepsy & Excessive Daytime Sleepiness"**

**S1 Fig., Part C. Scree plot for sub-factors of Main Factor 3, "Substances & Sleep"**

Scree plot for the factor analysis of factor 3

0

0.5

1

1.5

2

3.5

3

4

0

1

2

3

4

5

Subfactor

**S1 Fig., Part D. Scree plot for sub-factors of Main Factor 4, "Sleep Disordered Breathing"**
