# Supplementary material for: Creation of a shortened version of the Sleep Disorders Questionnaire (SDQ)
Source: PLoS One. 2024 Feb 6;19(2):e0288216. doi: 10.1371/journal.pone.0288216 (PMC10846718; doi:10.1371/journal.pone.0288216)
Supplement: S1 Table — (DOCX) [file pone.0288216.s002.docx]

**S1 Table: Content description of factors 5 – 19**

| **Main Factor Number^a^** | **Items loading on factor at**  > **0.4 (descending order)** | **Subject matter of the items** |
| --- | --- | --- |
| 5 | 95, 96, 97, 92, 94,  93, 102, 101, 99 | Family or childhood history of sleep and / or psychiatric problems. |
| 6 | 30, 35, 29 | Psychosomatic insomnia. |
| 7 | 77, 53, 28 | Epilepsy, seizures, and parasomnias. |
| 8 | 85, 84, 83, 79, 128, 90 | Unhappy intimate relationship |
| 9 | 85. 79. 84, 80, 83 | Sexual / intimacy problems |
| 10 | 47, 49, 46, 57 | Sleepiness in childhood |
| 11 | 113, 112, 119, 53 | Use of OTC drugs to facilitate sleep or wake |
| 12 | 170, 166, 108, 167, 168, 169 | Nicotine and caffeine usage. |
| 13 | 72, 74, 75, 70 | Tonsil / adenoid problems; head injury |
| 14 | 161, 162, 134 | Naps or rests are refreshing. |
| 15 | 130, 8, 34, 133, 6 | Stress and anxiety cause initial insomnia. |
| 16 | 124, 160 | Napping & employment |
| 17 | (merged with factor 12) | Usage of caffeinated drinks. |
| 18 | 125, 136, 126 | Sleepiness due to shift work. |
| 19 | 122, 123 | Happy family in childhood |

**S1 Table Legend:**

^a^ Main Factor Numbers relate to Fig. 1 in the main article. All of these factors emerged from the EFA with eigenvalues >1.00, but will not be included in the final SDQ-2. However, they may be useful for researchers in settings where diagnosis of clinical sleep disorders is not the focus.
