# Supplementary material for: Creation of a shortened version of the Sleep Disorders Questionnaire (SDQ)
Source: PLoS One. 2024 Feb 6;19(2):e0288216. doi: 10.1371/journal.pone.0288216 (PMC10846718; doi:10.1371/journal.pone.0288216)
Supplement: S2 Table — (DOCX) [file pone.0288216.s003.docx]

**S2 Table: Look-up matrix to estimate BMI from height (item 172) & weight (item 163) on the *original* SDQ**

|  | **Hgt =>** | **1** | **2** | **3** | **4** | **5** |
| --- | --- | --- | --- | --- | --- | --- |
| **Wgt.** |  |  |  |  |  |  |
| **1** |  | 24.06 | 22.48 | 20.21 | 18.88 | 18.36 |
| **2** |  | 26.39 | 24.66 | 22.17 | 20.71 | 20.14 |
| **3** |  | 30.79 | 28.77 | 25.86 | 24.16 | 23.50 |
| **4** |  | 35.28 | 32.96 | 29.63 | 27.68 | 26.93 |
| **5** |  | 37.71 | 35.23 | 31.67 | 29.59 | 28.78 |

**S2 Table Legend:**

Use this table to calculate an estimated Body Mass Index (BMI) from the respondent’s numeric answers to item 172 (height) to select the column and item 163 (weight) to select the row. The

estimated BMI is shown where the chosen row and column intersect. *Below is the matrix to use*

*if you are scoring the SDQ-2*.

-------

**Look-up matrix to estimate BMI from height (item 59) & weight (item 60) on the *SDQ-2***

|  | **Hgt =>** | **0** | **1** | **2** | **3** | **4** |
| --- | --- | --- | --- | --- | --- | --- |
| **Wgt.** |  |  |  |  |  |  |
| **0** |  | 24.06 | 22.48 | 20.21 | 18.88 | 18.36 |
| **1** |  | 26.39 | 24.66 | 22.17 | 20.71 | 20.14 |
| **2** |  | 30.79 | 28.77 | 25.86 | 24.16 | 23.50 |
| **3** |  | 35.28 | 32.96 | 29.63 | 27.68 | 26.93 |
| **4** |  | 37.71 | 35.23 | 31.67 | 29.59 | 28.78 |
