# Supplementary material for: Creation of a shortened version of the Sleep Disorders Questionnaire (SDQ)
Source: PLoS One. 2024 Feb 6;19(2):e0288216. doi: 10.1371/journal.pone.0288216 (PMC10846718; doi:10.1371/journal.pone.0288216)
Supplement: S1 Appendix — (DOCX) [file pone.0288216.s004.docx]

**S1 Appendix: Collinearity Diagnostics of Main Factors**

A collinearity problem occurs when a component associated with a high condition index contributes strongly (variance proportion greater than 0.5) to the variance of two or more items. Each factor was assessed for this problem.

In **Factor 1** **(Insomnia)**, there were no collinearity problems.

In **Factor 2** **(Narcolepsy / Daytime Sleepiness)**, there were *two pairs of collinear items:* *Item 55* (“I am very sleepy during the day and struggle to stay awake”) and *item 68* (“I am excessively sleepy during the daytime”). These share a high association (0.813, 0 .593 respectively) with component 25 (condition index 22.585). Item 55 will be kept because “struggling to stay awake” is a more useful description of daytime sleepiness than “excessively sleepy.” Item 68 is redundant and will be discarded.

*Item 62* (“I am often unable to move when I wake up in the morning”) and *item 39* (“I am unable to move after a nap”) are both highly associated (0.707 and 0.706) with component 22 (condition index 15.648). Item 39 will be kept because it is a better-written item, does not contain qualifiers like “often”, and is also suggestive of REM sleep during daytime naps. Item 62 will be discarded.

In **Factor 3 (Substances)**, there were two pairs of collinear items: *Item 105* (“I use alcohol to get to sleep”) and *item 106* (“I use alcohol to steady my nerves”). Both share a high association (0.653 and 0.681) with component 9 (condition index of 11.253). These items are likely redundant. Item 105 will be kept as it is more appropriate for a questionnaire about sleep disorders.

*Item 165* (“weight at age 20”) and *item 172* (“height”) share high associations (0.509 and 0.602) with component 10 (condition index of 11.479). The collinearity here is a reflection of a real association between two different variables. Since the condition index is not very high and these two items measure different, relevant things, both will be kept.

In **Factor 4** **(Sleep Disordered Breathing)**, there were two pairs of collinear items: *Item 21* (“I snore loudly enough to bother others”) and *item 20* (“I snore in my sleep”); these share a high association (0.807 and 0.852) with component 16 (condition index of 23.575). Item 21 will be kept as it is also a measure of the severity of the snoring (i.e., loud enough that it bothers other sleepers nearby). Item 20 will be discarded as redundant.

*Item 163* (“current weight”) and *item* *164* (“weight 6 months ago”) shared a high association (0.914 and 0.771) with component 18 (condition index of 40.687). The questions regarding weight six months ago and weight at age 20 (items 164 and 165) were included based on the observation that apnea often leads to weight gain. Weight gain since age 20 loads significantly on to the SA factor (loading of 0.564) but no significant correlation was found between SA and weight gain in the past six months (loading of 0.037). Item 164 can be discarded. Item 165 will be altered to directly ask about weight gain since age 20.
