# Supplementary material for: Creation of a shortened version of the Sleep Disorders Questionnaire (SDQ)
Source: PLoS One. 2024 Feb 6;19(2):e0288216. doi: 10.1371/journal.pone.0288216 (PMC10846718; doi:10.1371/journal.pone.0288216)
Supplement: S1 Questionnaire — (PDF) [file pone.0288216.s005.pdf]

Patient Name	Date of Birth
Today's Date	Doctor’s Name / Clinic Number

SDQ-2©

Sleep Disorders Questionnaire, 2nd Edition©

Version 2.2 (Dec. 2023)

created by

Alan Douglass MD[1,2] & Kathleen Biard PhD[3]

Copyright © 2010, 2023 by Alan B. Douglass
All rights reserved worldwide, including rights of translation.

SDQ-2© is a revised, reworded, and shortened version of the Sleep Disorders Questionnaire (SDQ© 1994), which was itself selected from a larger pool of questions that had been created at Stanford University Sleep Disorders Center by Drs. Laughton Miles, Christian Guilleminault, Vincent P. Zarcone Jr., and William C. Dement. The original SDQ© 1994 was copyrighted by Drs. A. Douglass, R. Bornstein, G. Nino-Murcia, S. Keenan, and the above Stanford authors.

REQUESTS FOR COPIES: Limited permission to copy SDQ-2 is granted to individual clinicians who will administer it to their own patients / clients. All other uses such as: research, posting online, re-publishing, or selling copies require written copyright clearance. Contact email: SDQ2@theroyal.ca and use subject heading “SDQ-2 copyright request”.

[1]The Royal’s Institute of Mental Health Research (affiliated with the University of Ottawa), 1145 Carling Ave., Ottawa, ON, Canada, K1Z 7K4
[2]Dept. of Psychiatry, University of Ottawa, Ontario, Canada
[3]School of Psychology, University of Ottawa, Ontario, Canada.

Instructions:

This questionnaire will give your doctor a good understanding about your problems with sleeping and waking. It is very important to answer every question, because some disorders show up as a pattern of answers to different questions.

In answering the questions, consider each question as applying to the ***past six months*** of your life, unless you have been told differently by the person who gave you this booklet.

Some people work night shift, or rotating shifts. Others have a very changeable bedtime. For these people, questions which ask about "day, daytime, morning, etc." will mean the time when they wake from their longest sleep of the day and become active. Similarly, "night, nighttime, bedtime, nocturnal" would refer to whenever they are having their longest sleep of the day.

Most of the questions are simple statements. You answer by circling a number from 0 to 4. If you strongly disagree with the statement, or if it never happens to you, answer "0". If the statement is always true in your case, or you agree strongly with it, answer "4". You may also choose "1 rarely", "2 sometimes", or "3 usually" as your answer. Notice that an *answer key* appears at the bottom of each page to remind you what is meant by the numbers. Please answer all of the questions.

Here is an example of how to fill out a question:

1. How often does it snow in Florida in July? (0) 1 2 3 4

IF YOU ARE CERTAIN THAT A QUESTION DOES NOT APPLY TO YOU, LEAVE IT BLANK. But . . . try to answer every question if at all possible. This is important. Notice that answer "0" can mean that the things asked in the question ***never*** happen to you.

If you are using the computerized answer sheet, blacken the space which corresponds to your answer, "0 to 4", instead of circling the answer in this booklet.

1. I have trouble getting to sleep at night	0 1 2 3 4
2. Now, I am sleepy during the day and I struggle to stay awake	0 1 2 3 4
3. I have a poor night's sleep	0 1 2 3 4
4. I am told I snore loudly and bother others	0 1 2 3 4
5. After waking at night, I fear I will not be able to get back to sleep	0 1 2 3 4
6. I now have trouble doing my job because of sleepiness or fatigue	0 1 2 3 4
7. I use alcohol in order to get to sleep	0 1 2 3 4
8. My night sleep is restless and disturbed	0 1 2 3 4
9. I have to let someone else drive the car because I am too sleepy to do it	0 1 2 3 4
10. At bedtime, I'm afraid of not being able to go to sleep	0 1 2 3 4
11. I am very sleepy in the daytime, and this seems to go in cycles at regular intervals	0 1 2 3 4
12. I have used "street drugs" (marijuana, "uppers", "downers", narcotics, hallucinogens, cocaine)	0 1 2 3 4
13. I get too little sleep at night	0 1 2 3 4
14. I have slept for several days at a time, or at least I have been overwhelmingly sleepy for that long	0 1 2 3 4
15. I feel that I have insomnia	0 1 2 3 4
16. In the past 6 months, I have fallen asleep accidentally in some of these situations: eating a meal, talking on the phone, talking to someone, riding in a bus or car, watching TV, at a theater, reading a book, at a lecture.	0 1 2 3 4
17. While drinking alcohol, I have carried out actions without being aware of them, and not remembered them the next day	0 1 2 3 4
18. My sleep is disturbed by fears that I might not be able to get back to sleep if I should wake up	0 1 2 3 4
19. My sleep is disturbed by thoughts racing through my mind	0 1 2 3 4

** Key for answers ***

0	1	2	3	4
NEVER	RARELY	SOMETIMES	USUALLY	ALWAYS
(strongly disagree)	(disagree)	(not sure)	(agree)	(agree strongly)

20. When falling asleep, I have "restless legs" (a feeling of crawling, aching, or inability to keep legs still)	0 1 2 3 4
21. I have used marijuana to help me go to sleep	0 1 2 3 4
22. I have a problem with my sleep	0 1 2 3 4
23. I have vivid dreams during my daytime naps	0 1 2 3 4
24. I smoke tobacco within two hours of bedtime	0 1 2 3 4
25. My sleep is disturbed by worrying about things	0 1 2 3 4
26. I find myself doing things which make no sense, such as writing nonsense instead of notes, or mixing together chocolate and gravy	0 1 2 3 4
27. I am told I stop breathing ("hold my breath") in sleep	0 1 2 3 4
28. I wake up during the night	0 1 2 3 4
29. I got bad grades in school because I was too sleepy	0 1 2 3 4
30. I have used tobacco to help me go to sleep	0 1 2 3 4
31. At bedtime, I worry about things	0 1 2 3 4
32. I get sudden muscular weakness (or even a brief period of paralysis, being unable to move) when laughing, angry, or in situations of strong emotion	0 1 2 3 4
33. At bedtime, thoughts race through my mind	0 1 2 3 4
34. Sometimes I realize I have driven my car to the wrong place, and I can't remember how I did it	0 1 2 3 4
35. My sleep is disturbed by sadness or depression	0 1 2 3 4
36. I feel unable to move (paralyzed) after a nap	0 1 2 3 4
37. My snoring or my breathing problem is worse if I sleep on my back	0 1 2 3 4
38. At bedtime, I feel sad and depressed	0 1 2 3 4
39. I am sleeping more than I used to	0 1 2 3 4
40. Mental stress, worry, or anxiety worsens my sleep	0 1 2 3 4

*** Key for answers ***

0	1	2	3	4
NEVER	RARELY	SOMETIMES	USUALLY	ALWAYS
(strongly disagree)	(disagree)	(not sure)	(agree)	(agree strongly)

41. I get "weak knees" when I laugh	0 1 2 3 4
42. I awaken suddenly, gasping for breath, unable to breathe	0 1 2 3 4
43. At night, my sleep disturbs my bed partner's sleep	0 1 2 3 4
44. At bedtime, I feel muscular tension	0 1 2 3 4
45. I see vivid dream-like images (hallucinations) either just before or just after a daytime nap, yet I am sure I am awake when they happen	0 1 2 3 4
46. I have been unable to sleep <u>at all</u> for several days	0 1 2 3 4
47. I have dream-like images (hallucinations) when I awaken in the morning even though I know I am not asleep	0 1 2 3 4
48. My snoring or my breathing problem is worse if I fall asleep right after drinking alcohol	0 1 2 3 4
49. My sleep is disturbed by muscular tension	0 1 2 3 4
50. When falling asleep, I feel paralyzed (unable to move)	0 1 2 3 4
51. I have high blood pressure (or once had it)	0 1 2 3 4
52. My snoring or my breathing problem is worse when I have an allergy or infection in the nose, throat, or chest	0 1 2 3 4
53. I take a prescription drug which the doctor gave me mainly to help me sleep (sleeping pills, anti-depressants, tranquilizers)	0 1 2 3 4
54. I have a problem with my nose blocking up when I am trying to sleep (allergies, infections)	0 1 2 3 4
55. My sleep is disturbed by "restless legs" (a feeling of crawling, aching, inability to keep legs still)	0 1 2 3 4
56. I feel that my sleep is abnormal	0 1 2 3 4
57. <u>FOR WOMEN ONLY:</u> I have gone through menopause ("change of life")	0 1 2 3 4

** Key for answers **

0	1	2	3	4
NEVER	RARELY	SOMETIMES	USUALLY	ALWAYS
(strongly disagree)	(disagree)	(not sure)	(agree)	(agree strongly)

IN THIS SECTION, PLEASE CIRCLE THE ITEM (NUMBERED 0--4) WHICH BEST MATCHES YOUR ANSWER.

---

58. How old are you now?
    0.) 25 or under   1.) 26-35 yr.   2.) 36-44 yr.
    3.) 45–50 yr.   4.) 51 yr. or older

59. How tall are you?
    0.) 5'3" (160 cm) or less   1.) 5'4" -- 5'6.5" (161–169 cm)
    2.) 5'7" -- 5'9.5" (170–177 cm)   3.) 5'10" -- 5'11.5" (178–181 cm)
    4.) 6'0" (182 cm) or taller

60. What is your current weight?
    0.) 134 lb. (61 kg) or less   1.) 135–159 lb. (62–72 kg)
    2.) 160–183 lb. (73–83 kg)   3.) 184–209 lb. (84–95 kg)
    4.) 210 lb. (96 kg) or more

61. How much weight have you gained since age 20?
    0.) None, or have lost weight   1.) 1–20 lb. (1–9 kg)   2.) 21–39 lb. (10–18 kg)
    3.) 40–80 lb. (19–36 kg)   4.) 81 lb. (37 kg) or more

62. How long is your longest wake period at night?
    0.) Less than 5 min.   1.) Six to 19 min.   2.) 20 to 59 min.
    3.) One to 2 hrs.   4.) More than 2 hrs.

63. How many hours of sleep do you get at night, not including time spent awake in bed?
    0.) Eight or more   1.) Seven hrs.   2.) Six hrs.
    3.) Four to 5 hrs.   4.) Less than 4 hrs.

64. How many daytime naps (asleep for 5 minutes or more) do you take on an average working day?
    0.) None   1.) One   2.) Two
    3.) Three or four   4.) Five or more

65. How many car accidents or "near misses" have you had because of excessive sleepiness?
    0.) None   1.) One   2.) Two
    3.) Three   4.) Four or more

66. Please indicate your sex at birth: Male ____ Female ____

=== END ===
