# Supplementary material for: Creation of a shortened version of the Sleep Disorders Questionnaire (SDQ)
Source: PLoS One. 2024 Feb 6;19(2):e0288216. doi: 10.1371/journal.pone.0288216 (PMC10846718; doi:10.1371/journal.pone.0288216)
Supplement: S1 File — (DOCX) [file pone.0288216.s006.docx]

**SUBSCALE SCORING SHEET FOR SDQ-2**

**Subscales include:** *InsPsy* (psychological features of insomnia), *InsSubj* (subjective estimate of insomnia severity), *InsPLM* (insomnia due to limb movements), *EDS* (excessive daytime sleepiness), *NAR* (neurological symptoms of narcolepsy), *SUBST* (sleep effects of substance use), and *SDB* (sleep-disordered breathing).

Copy the respondent’s numerical answers to the questions (“Q#”) into the slots under subscale name; add the columns for subscale totals.

| **Q#** | **InsPsy** |  | **Q#** | **InsSubj** |  | **Q#** | **InsPLM** |  | **Q#** | **EDS** |
| --- | --- | --- | --- | --- | --- | --- | --- | --- | --- | --- |
| 1 |  |  | 3 |  |  | 20 |  |  | 2 |  |
| 5 |  |  | 8 |  |  | 44 |  |  | 6 |  |
| 10 |  |  | 13 |  |  | 49 |  |  | 9 |  |
| 18 |  |  | 15 |  |  | 55 |  |  | 11 |  |
| 19 |  |  | 22 |  |  | TOTAL = |  |  | 14 |  |
| 25 |  |  | 28 |  |  |  |  |  | 16 |  |
| 31 |  |  | 46 |  |  |  |  |  | 39 |  |
| 33 |  |  | 53 |  |  |  |  |  | 64 |  |
| 35 |  |  | 56 |  |  |  |  |  | 65 |  |
| 38 |  |  | 62 |  |  |  |  |  | TOTAL = |  |
| 40 |  |  | 63 |  |  |  |  |  |  |  |
| TOTAL = |  |  | TOTAL = |  |  |  |  |  |  |  |
|  |  |  |  |  |  |  |  |  |  |  |
|  |  |  |  |  |  |  |  |  |  |  |
|  |  |  |  |  |  |  |  |  |  |  |
| **Q#** | **NAR** |  | **Q#** | **SUBST** |  | **Q#** | **SDB** |  |  |  |
| 23 |  |  | 7 |  |  | 4 |  |  |  |  |
| 26 |  |  | 12 |  |  | 27 |  |  |  |  |
| 29 |  |  | 17 |  |  | 37 |  |  |  |  |
| 32 |  |  | 21 |  |  | 42 |  |  |  |  |
| 34 |  |  | 24 |  |  | 43 |  |  |  |  |
| 36 |  |  | 30 |  |  | 48 |  |  |  |  |
| 41 |  |  | TOTAL = |  |  | 51 |  |  |  |  |
| 45 |  |  |  |  |  | 52 |  |  |  |  |
| 47 |  |  |  |  |  | 54 |  |  |  |  |
| 50 |  |  |  |  |  | ** 57 |  |  |  |  |
| TOTAL = |  |  |  |  |  | 58 |  |  |  |  |
|  |  |  |  |  |  | 59 |  |  |  |  |
|  |  |  |  |  |  | 60 |  |  |  |  |
|  |  |  |  |  |  | 61 |  |  |  |  |
|  |  |  |  |  |  | TOTAL = |  |  |  |  |

**57 is the menopause question. If respondent is male, code it as MISSING

SDQ-2 item #63 (number of hours of sleep at night) is reverse-coded, so “0” means respondent reports 8 hours.
SDQ-2 item #66 does not appear on any scale; it is “sex at birth”, coded with “X” under “Male” or “Female”

**SUBSCALE SCORING SHEET FOR *the Original* *SDQ (1994)***

**Subscales include:** *InsPsy* (psychological features of insomnia), *InsSubj* (subjective estimate of insomnia severity), *InsPLM* (insomnia due to limb movements), *EDS* (excessive daytime sleepiness), *NAR* (neurological symptoms of narcolepsy), *SUBST* (sleep effects of substance use), and *SDB* (sleep-disordered breathing).

Copy the respondent’s numerical answers to the questions (“Q#”) into the slots under subscale name; add the columns for subscale totals.

| **Q#** | **InsPsy** |  | **Q#** | **InsSubj** |  | **Q#** | **InsPLM** |  | **Q#** | **EDS** |
| --- | --- | --- | --- | --- | --- | --- | --- | --- | --- | --- |
| 3 |  |  | 1 |  |  | 9 |  |  | 41 |  |
| 6 |  |  | 2 |  |  | 12 |  |  | 42 |  |
| 7 |  |  | 4 |  |  | 31 |  |  | 55 |  |
| 8 |  |  | 14 |  |  | 35 |  |  | 56 |  |
| 10 |  |  | 43 |  |  | TOTAL = |  |  | 58 |  |
| 13 |  |  | 44 |  |  |  |  |  | 59 |  |
| 32 |  |  | 45 |  |  |  |  |  | 91 |  |
| 33 |  |  | 87 |  |  |  |  |  | 157 |  |
| 34 |  |  | 114 |  |  |  |  |  | 158 |  |
| 36 |  |  | ##153 | ## |  |  |  |  | TOTAL = |  |
| 130 |  |  | 154 |  |  |  |  |  |  |  |
| TOTAL = |  |  | TOTAL = |  |  |  |  |  |  |  |
|  |  |  |  |  |  |  |  |  |  |  |
|  |  |  |  |  |  |  |  |  |  |  |
|  |  |  |  |  |  |  |  |  |  |  |
| **Q#** | **NAR** |  | **Q#** | **SUBST** |  | **Q#** | **SDB** |  |  |  |
| 11 |  |  | 105 |  |  | 15 |  |  |  |  |
| 39 |  |  | 107 |  |  | 21 |  |  |  |  |
| 40 |  |  | 108 |  |  | 22 |  |  |  |  |
| 57 |  |  | 109 |  |  | 23 |  |  |  |  |
| 60 |  |  | 110 |  |  | 71 |  |  |  |  |
| 61 |  |  | 111 |  |  | 139 |  |  |  |  |
| 63 |  |  | TOTAL = |  |  | 141 |  |  |  |  |
| 64 |  |  |  |  |  | 142 |  |  |  |  |
| 66 |  |  |  |  |  | 143 |  |  |  |  |
| 67 |  |  |  |  |  | **144 |  |  |  |  |
| TOTAL = |  |  |  |  |  | 163 |  |  |  |  |
|  |  |  |  |  |  | 165 |  |  |  |  |
|  |  |  |  |  |  | 172 |  |  |  |  |
|  |  |  |  |  |  | 173 |  |  |  |  |
|  |  |  |  |  |  | TOTAL = |  |  |  |  |

**144 is the menopause question. If respondent is male, code it as MISSING

SDQ item #153 (number of hours of sleep at night) needs to be reverse-coded, so “1 2 3 4 5” => “5 4 3 2 1”.
Female sex can be inferred if the respondent completed any of items #144 – 147.
