# Supplementary material for: Creation of a shortened version of the Sleep Disorders Questionnaire (SDQ)
Source: PLoS One. 2024 Feb 6;19(2):e0288216. doi: 10.1371/journal.pone.0288216 (PMC10846718; doi:10.1371/journal.pone.0288216)
Supplement: S1 Checklist — (DOCX) [file pone.0288216.s008.docx]

STROBE Statement—checklist of items that should be included in reports of observational studies

|  | Item No. | Recommendation | Page  No. | Relevant text from manuscript |
| --- | --- | --- | --- | --- |
| **Title and abstract** | 1 | (*a*) Indicate the study’s design with a commonly used term in the title or the abstract | 3 | Retrospective chart review |
|  |  | (*b*) Provide in the abstract an informative and balanced summary of what was done and what was found | 3 | “SDQ results from a retrospective review of the charts of 2131 persons from 7 sleep disorders clinics ” |
| Introduction | | | |  |
| Background/rationale | 2 | Explain the scientific background and rationale for the investigation being reported | 10 | “…the above findings suggest that the use of SDQ in non-clinical populations sometimes substantially over-diagnoses sleep pathologies. They also imply that some SDQ subscales are confounded with each other,” |
| Objectives | 3 | State specific objectives, including any prespecified hypotheses | 10 | “…reassess the factor structure of the 4 existing SDQ subscales via EFA. It was also hoped that new subscales could be derived, such as a specific insomnia subscale. Finally, it was hoped to reduce the total number of questions from 176 to a more manageable size …” |
| Methods | | | |  |
| Study design | 4 | Present key elements of study design early in the paper | 11 | “Any remaining missing values among the 176 items were replaced by using a multiple imputation procedure (“PROC-MI”, SAS version 9.1, SAS Institute, Cary, North Carolina) …. This version of SAS software was also used to compute the EFAs (“PROC-FACTOR”) using a varimax factor rotation of both orthogonal and oblique types. Eigenvalues and scree plots were used to determine the final number of factors to be retained.” |
| Setting | 5 | Describe the setting, locations, and relevant dates, including periods of recruitment, exposure, follow-up, and data collection | 10 | “SDQ responses of 2185 adults (1543 males, 642 females) were obtained for secondary analysis from a variety of hospitals and universities at which the SDQ had been completed prior to a laboratory nocturnal poly-somnogram, over the period 1986 – 2008” |
| Participants | 6 | (*a*) *Cohort study*—Give the eligibility criteria, and the sources and methods of selection of participants. Describe methods of follow-up  *Case-control study*—Give the eligibility criteria, and the sources and methods of case ascertainment and control selection. Give the rationale for the choice of cases and controls  *Cross-sectional study*—Give the eligibility criteria, and the sources and methods of selection of participants | 11 | Retrospective cross-sectional study of sleep clinic patients’ charts that contained a completed Sleep Disorders Questionnaire (SDQ), comprised of 176 questions |
|  |  | (*b*) *Cohort study*—For matched studies, give matching criteria and number of exposed and unexposed  *Case-control study*—For matched studies, give matching criteria and the number of controls per case |  | N/A |
| Variables | 7 | Clearly define all outcomes, exposures, predictors, potential confounders, and effect modifiers. Give diagnostic criteria, if applicable | 11 | { patients of all psychiatric and sleep-disorder diagnoses were accepted into the study } |
| Data sources/ measurement | 8* | For each variable of interest, give sources of data and details of methods of assessment (measurement). Describe comparability of assessment methods if there is more than one group | 11 | { all data consisted of self-report questionnaire items answered on a Likert scale } |
| Bias | 9 | Describe any efforts to address potential sources of bias | 11 | { nearly all participants were hospital inpatients or outpatients, so this study does not reflect the general population } |
| Study size | 10 | Explain how the study size was arrived at | 11 | “The data from 54 cases (2.5% of total) were discarded because more than 20% of items were unanswered, leaving the data of 2131 persons (1497 males, 634 females) for the EFA. This gave a patient-to-questionnaire-item ratio of 12:1, which is commonly regarded as adequate for multivariate analysis.” |

Continued on next page

| Quantitative variables | 11 | Explain how quantitative variables were handled in the analyses. If applicable, describe which groupings were chosen and why |  | { see answer to item #4 } |
| --- | --- | --- | --- | --- |
| Statistical methods | 12 | (*a*) Describe all statistical methods, including those used to control for confounding |  | { see answer to item #4 } |
|  |  | (*b*) Describe any methods used to examine subgroups and interactions | 11 | Males & females were first analyzed together and then later separately, as a sensitivity analysis. |
|  |  | (*c*) Explain how missing data were addressed |  | { see answer to item #4 } |
|  |  | (*d*) *Cohort study*—If applicable, explain how loss to follow-up was addressed  *Case-control study*—If applicable, explain how matching of cases and controls was addressed  *Cross-sectional study*—If applicable, describe analytical methods taking account of sampling strategy | 10 | “These data therefore represented a sample of convenience and not a population sample planned in advance.” |
|  |  | (*e*) Describe any sensitivity analyses |  | { see answer to #12b } |
| Results | | | | |
| Participants | 13* | (a) Report numbers of individuals at each stage of study—eg numbers potentially eligible, examined for eligibility, confirmed eligible, included in the study, completing follow-up, and analysed |  | { see answer to #10 } |
|  |  | (b) Give reasons for non-participation at each stage |  | { see answer to #10 } |
|  |  | (c) Consider use of a flow diagram |  |  |
| Descriptive data | 14* | (a) Give characteristics of study participants (eg demographic, clinical, social) and information on exposures and potential confounders | Pg.  12 | \|  \|  \| **MALES** \|  \|  \|  \| **FEMALES** \|  \| \| --- \| --- \| --- \| --- \| --- \| --- \| --- \| --- \| \| **DIAGNOSTIC GROUP** \| **N** \| **Mean Age** \| **SD** \|  \| **N** \| **Mean Age** \| **SD** \| \| Psychiatric Patients^a^ \| 574 \| 45.18 \| 13.41 \|  \| 296 \| 41.85 \| 12.67 \| \| General Practice Referrals^b^ \| 899 \| 45.23 \| 13.77 \|  \| 338 \| 44.78 \| 13.79 \| \| Normal control subjects \| 70 \| 38.70 \| 12.71 \|  \| 8 \| 38.61 \| 12.73 \| \| **SUBTOTALS** \| **1543** \| **44.92** \| **13.59** \|  \| **642** \| **43.35** \| **13.27** \| \| **Male + Female TOTAL** \|  \|  \|  \| **2185** \|  \|  \|  \| \| 54 **removed (missing data)** \| **1497** \|  \|  \| **2131** \| **634** \|  \|  \| |
|  |  | (b) Indicate number of participants with missing data for each variable of interest | 12 | “Fifty-four cases were deleted due to missing data, giving a case-wise missing rate of 2.5%. After deletions, the remaining variable-wise average missing rate was 3.1%” |
|  |  | (c) *Cohort study*—Summarise follow-up time (eg, average and total amount) |  | N/A |
| Outcome data | 15* | *Cohort study*—Report numbers of outcome events or summary measures over time |  | N/A |
|  |  | *Case-control study—*Report numbers in each exposure category, or summary measures of exposure |  | N/A |
|  |  | *Cross-sectional study—*Report numbers of outcome events or summary measures | 14 | “Secondary EFA of Main Factor 1 resulted in 3 subfactors with clinical relevance, while the same process on Main Factor 2 resulted in 2 subfactors. Therefore, 7 useful factors were extracted from the SDQ data.” |
| Main results | 16 | 1. Give unadjusted estimates and, if applicable, confounder-adjusted estimates and their precision (eg, 95% confidence interval). Make clear {. . . cont’d} 2. which confounders were adjusted for and why they were included | 13 | \| **Factor^a^** \| **Orthogonal Rotation** \| **Oblique Rotation** \| \| \| --- \| --- \| --- \| --- \| \|  \|  \| Shared variance \| Unique variance \| \| 1 \| 12.76 \| 15.43 \| 8.92 \| \| 2 \| 8.89 \| 11.53 \| 6.69 \| \| 3 \| 6.68 \| 8.67 \| 5.91 \| \| 4 \| 6.57 \| 6.46 \| 6.43 \|   **TOTAL 34.90 42.09 27.95**  {confounding effects of variables not selected for a given factor were removed by the exploratory factor analysis calculations} |
|  |  | (*b*) Report category boundaries when continuous variables were categorized |  | N/A |
|  |  | (*c*) If relevant, consider translating estimates of relative risk into absolute risk for a meaningful time period |  | N/A |

Continued on next page

| Other analyses | 17 | Report other analyses done—eg analyses of subgroups and interactions, and sensitivity analyses |  | {see answer to 12b} |
| --- | --- | --- | --- | --- |
| Discussion | | | | |
| Key results | 18 | Summarise key results with reference to study objectives | 23 & 24 | “confirmed and resolved problems with the 4 subscales of the original SDQ, notably their conflation of general daytime sleepiness with neurological symptoms of narcolepsy, and a similar conflation of psychiatric insomnia symptoms with those of simple insomnia disorder.”  “In addition, the newly extracted factors comprised only 66 items, allowing SDQ-2 to be much quicker to administer than the original questionnaire.” |
| Limitations | 19 | Discuss limitations of the study, taking into account sources of potential bias or imprecision. Discuss both direction and magnitude of any potential bias | 26 & 27 | “Nevertheless, there are some cautionary notes. The sample of SDQ respondents upon which the present study is based was not a random population sample. Most were either referred to sleep disorder clinics with a suspected sleep disorder or were patients with another known disorder that was suspected of causing sleep-wake symptoms, i.e., depression, schizophrenia, alcoholism, chronic fatigue syndrome, or general medical illness.” |
| Interpretation | 20 | Give a cautious overall interpretation of results considering objectives, limitations, multiplicity of analyses, results from similar studies, and other relevant evidence | 28 | “The new SDQ-2 should have more clinical utility than the original SDQ, in that it identifies a greater range of sleep pathologies and does it with better precision.” |
| Generalisability | 21 | Discuss the generalisability (external validity) of the study results | 28 | “A prospective study of 800 members of a large urban police force has been completed, in which the 7 new SDQ-2 subscales were concurrently validated against {9} published sleep and psychological questionnaires” |
| Other information | |  | | |
| Funding | 22 | Give the source of funding and the role of the funders for the present study and, if applicable, for the original study on which the present article is based | 2 | Canadian Government NSERC grant to PhD candidate Biard for the analysis |

*Give information separately for cases and controls in case-control studies and, if applicable, for exposed and unexposed groups in cohort and cross-sectional studies.

**Note:** An Explanation and Elaboration article discusses each checklist item and gives methodological background and published examples of transparent reporting. The STROBE checklist is best used in conjunction with this article (freely available on the Web sites of PLoS Medicine at http://www.plosmedicine.org/, Annals of Internal Medicine at http://www.annals.org/, and Epidemiology at http://www.epidem.com/). Information on the STROBE Initiative is available at www.strobe-statement.org.
