# Supplementary material for: Creation of a shortened version of the Sleep Disorders Questionnaire (SDQ)
Source: PLoS One. 2024 Feb 6;19(2):e0288216. doi: 10.1371/journal.pone.0288216 (PMC10846718; doi:10.1371/journal.pone.0288216)
Supplement: Attachment — Submitted filename: REBUTTAL LETTER.docx [file pone.0288216.s009.docx]

Date: Sept. 15, 2023

Mohammad Hossein Ebrahimi

Academic Editor, PLOS ONE

<< Submitted with manuscript to website >>

Dear Sir:

RE: **Creation of a Shortened Version of the of the Sleep Disorders Questionnaire (SDQ)**

**[PONE-D-23-17429] - [EMID:9eef891b3c87a3df]**

This letter is my rebuttal to the issues raised by the peer reviewers of this MS as it was previously submitted. I am happy to say that there were very few such issues, and all have been fully resolved in the final draft of the MS.

The issues were:

1. That PLOS Data policy “requires authors to make all data underlying the findings described in their manuscript fully available without restriction”. *The full 167 variables on 2131 subjects are now uploaded with the MS as a large Excel file, with missing data imputed by MICE.*
2. “It might be better if the whole set of questions in SDQ-2 is shown in this paper”. *A fully-formatted printed version of the SDQ-2, version 2.1 is now uploaded with the MS, along with a scoring page so that users can reduce the raw responses into the 7 subscale scores.*

In closing, I thank the two peer reviewers for their careful reading of the MS and I thank them for their suggestions, which certainly will improve the published paper.

Yours sincerely,

Alan B. Douglass, MD,
FRCPC (psychiatry), Dip Amer Board of Sleep Medicine

Dip Amer Board of Psychiatry and Neurology

Physician Scientist, University of Ottawa’s IMHR
